# A Screen-Printed Metal Hybrid Composite for Wireless Wind Sensing

**DOI:** 10.3390/nano12060972

**Published:** 2022-03-15

**Authors:** Xue Qi, Sooman Lim

**Affiliations:** Department of Flexible and Printable Electronics, LANL-JBNU Engineering Institute, Jeonbuk National University, Jeonju 54896, Korea; qixuerhea@jbnu.ac.kr

**Keywords:** screen-printed wind sensor, rheology, gauge factor, temperature, wireless sensor

## Abstract

Wind sensing has become a key component in various fields with the growing trend of assessing air conditions for energy conversion. In this study, we demonstrated a wireless screen-printable flexible strain sensor system based on Ag/MWCNT composite for wind sensing. To achieve high printability with the metal hybrid composite for the fabrication of a screen-printed flexible sensor, we systematically investigated the rheological properties, resulting in the high shear thinning and thixotropic behavior of the composite. After confirming the suitability for screen printing, we investigated the performance of the printed strain sensor, obtaining a gauge factor (G.F.) of 2.08 with 90% sensitivity and high durability after 6000 bending cycles. In addition, the sensor showed 98% temperature sensitivity during a wind sensing test due to the intrinsic properties of the metal hybrid composite. In an application based on an IoT system, we verified that the response of the wireless sensor corresponded with that of a wired sensor, indicating the expansion of low-cost, mass-produced screen-printed wind sensors.

## 1. Introduction

Wind sensing has been utilized in various fields, including in the determination of drying rates [1,2], pollen propagation [3,4,5], pathogen propagation [6,7,8], air conditioning strategy design [9,10,11], and wind turbine operations [12,13,14]. To measure wind velocity, traditional measuring mechanisms are widely used, such as vane and cup anemometers [15,16,17] and acoustic anemometers [18]. However, these mechanisms have detection thresholds due to mechanical wear, performance degradation, and bearing friction, and are relatively expensive, limiting their use [19]. Another measuring mechanism includes the use of a pressure anemometer, which works based on the measurement of barometric pressure proportional to the square of the wind velocity [20]. Although this approach can provide accurate wind velocity measurements, the tubes are prone to clogging by water droplets and ice crystals [21].

To avoid these defects, a low-cost, low-complexity, powerful wind direction sensor using 3D printed flexible strain sensor has been demonstrated [20]. In this previous work, conductive polylactic acid was printed on a flexible substrate via the fused deposition method.

The printing process can be modeled so that core and adjustable design aspects can be fitted to specific wind sensing applications. However, due to the nature of the FDM printing process, the low printing speeds and limited productivity are still problematic. Alternatively, screen printing is the most widely used process for the fabrication of flexible strain sensors because of its advantages of fast production, massive productivity, easy design, and less limited surface morphology of the substrates [22,23,24]. However, the screen printing process has not been applied for wind sensing applications in various fields.

When applying printed flexible strain sensors for wind sensing, the conductive material is an important component, since it mainly determines the sensing properties. Carbon nanotubes (CNTs) have been broadly used in strain sensors as conductive materials due to their excellent mechanical properties, chemical inertness, and low cost [25,26,27]. To make up for certain defects that carbon base materials often suffer from, such as low conductivity (sheet resistance of ≈4 KΩ sq^−15^) and relatively low sensitivity, researchers have mixed CNTs with other metal materials. A previous study physically mixed CNTs with silver particles [28], while another using chemical methods produced AgNP-modified CNTs [29], resulting in improved electrical properties and a high gauge factor (GF) sensitivity with the results being two times better than for CNTs. Conventionally, strain sensor networks require wires to connect each individual strain sensor to a centralized data acquisition unit; however, this causes measurement limitations, high maintenance costs, and reductions in spatial resolution [30,31,32]. Thus, the fabrication of wireless sensors with a wireless communication channel is preferred and expected to offer unique advantages, such as installation flexibility, reduced weight, improved sensor density, and ease of maintenance [33,34,35]. On the other hand, simulations are being used in various fields to analyze specific phenomena [36,37]. However, the sensing mechanism is still poorly understood. In particular, the calculation of strain energy via the piezo resistance and applied load plays a key role in this study.

In this study, a wireless screen-printable flexible strain sensor system based on an Ag/MWCNT composite was designed for wind sensing. The sensor’s fast production speed, high productivity, simple design, low cost, stability, and lower restrictions on the surface morphology of the substrate mean it has wider application prospects. To use the optimized Ag/MWCNT composite for the fabrication of a screen-printed flexible sensor, we investigated the rheological properties of the composite to verify the printability of the synthesized ink, whereby the performance of the strain sensor revealed a gauge factor (G.F.) of 2.08 with 90% sensitivity and high durability after 6000 bending cycles. In addition, the sensor showed 98% temperature sensitivity during a wind sensing test due to the intrinsic properties of the metal hybrid composite. Finally, the printed sensor was proven to offer greater performance in wireless systems compared to that of wired systems. Compared to other recent results that have been published (Table 1), the wireless screen-printable flexible strain sensor system based on Ag/MWCNT composite for wind sensing showed excellent performance in terms of its stability, applicability, simple production process, and low substrate limits.

## 2. Experiment

### 2.1. Materials

AgNPs were purchased from NTbase (South Korea), and the model of AgNP was NP-S80, with a purity of 99.99%. The OD values of MWCNTs (US Research Nanomaterials, Inc., Houston, TX, USA) were 5–15 nm, with a purity of >95%. The fluorosurfactant (FC-4430) was obtained from 3M United States. Sterocoll^®^ HS, FoamStar^®^ SI 2213 (St. Paul, MN, USA) and Hdropal-at^®^ WE 3475 were provided by BASF corporation (Florham Park, NJ, USA). Butyl carbitol and PVB terpolymers were purchased from Sigma-Aldrich (Burlington, MA, USA).

### 2.2. Preparation of Ag/MWCNT Composite

**Figure 1:**
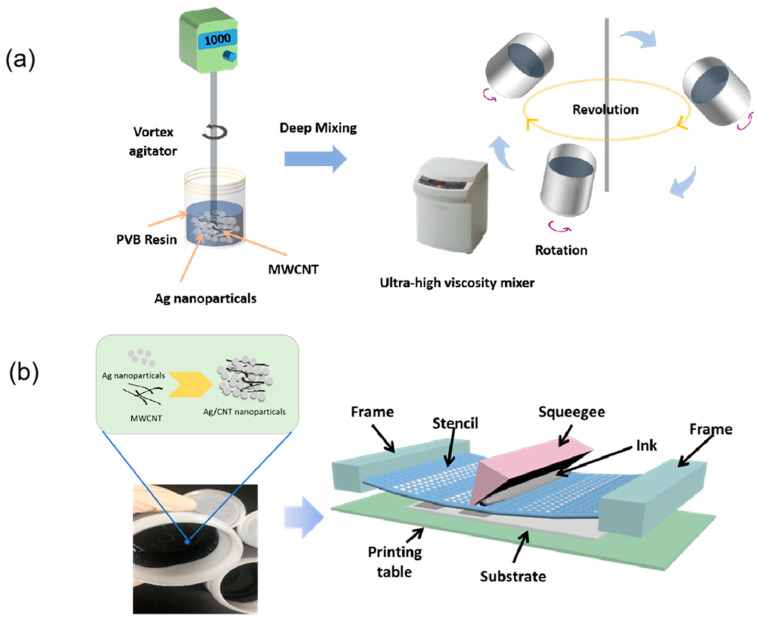
(**a**) Preparation process of thermally conductive silver nanoparticle MWCNT composites. (**b**) A schematic diagram of the screen printing process with Ag/MWCNT composite for the fabrication of a wind sensor.

As shown in Figure 1a, a simple, low-cost, room temperature chemical solution process was used to prepare the Ag/CNT paste. The ratio of the Ag/CNT, dispersant (FC-4430), solvent–resin vehicle (Butyl carbitol/PVB—3.75:21.25), thicker (Sterocoll^®^ HS), defoaming agent (FoamStar^®^ SI 2213), and wetting agent (Hydropalat^®^ WE 3475) was 2.4:47.7:0.1:47.7:1:0.1:1. The materials were mixed using a vortex agitator (Daihan Scientific, Wonju, South Korea) at 1000 RPM for 12 h. The paste was transferred to a planetary centrifugal bubble-free mixer (ARE-310, THINKY U.S.A., Inc., Laguna Hills, CA, USA) for 20 min, then degassed for 5 min, and this process was repeated three times.

### 2.3. Fabrication of Printed Strain Sensor with Screen Printing

Screen printing of the composite was performed on an AMX-1242T Semi-Auto screen printer (Korea), using a 320 mm × 320 mm precision stainless steel screen mesh (400 mesh count, 0.84 mm wire diameter, 0.83 mm mesh opening, standard mesh tension, 39 µm mesh thickness, 10 µm emulsion thickness, emulsion is safe with water) from Samborn Screens (Korea). A 140 mm squeegee with an ergonomic holder forming an angle of approximately 45° was used to print a pattern with a printing velocity of 30 mms^−1^. The stretchable film was fixed on the screen printing substrate and configured Ag/MWCNT composite loaded on one end of the mesh-screen, and then the composite was transferred to the substrate film through squeezing and drying for 30 min at 120 °C.

### 2.4. Characterization

A mixed-flow instrument (hr-3TA Instruments, New Castle, DE, USA) was used to measure the viscosity of Ag/MWCNT composite. The current signal system voltage was 5.0 V (Keithley 2400 source meter, Beaverton, OR, USA), which was used to test the durability of the sensor by bending the system (OWIS PS 10-32, Staufen, Germany). The morphologies of composite films were analyzed using field-emission–scanning electron microscopy (SUPRA 40VP, Carl Zeiss, Germany) at the Center for University-Wide Research Facilities (CURF) at Jeonbuk National University.

## 3. Results and Discussion

Figure 1b shows the fabrication of a strain sensor fully printed with Ag/MWC-NT composite on a PEN substrate. The reason for using PEN as a substrate was due to its high tensile strength, excellent dimensional stability, low moisture absorption, and good retention of physical properties over a wide temperature range. For the screen printing method, the composite is placed on the screen, pulled with strong tension, and pressed with a squeegee (a spatula-shaped urethane rubber material). During the process, the screen printer maintains a snap-off distance between the screen and the substrate, and when the squeegee passes the screen meets the substrate, transfers the composite, and deposits it by releasing the tension.

**Figure 2:**
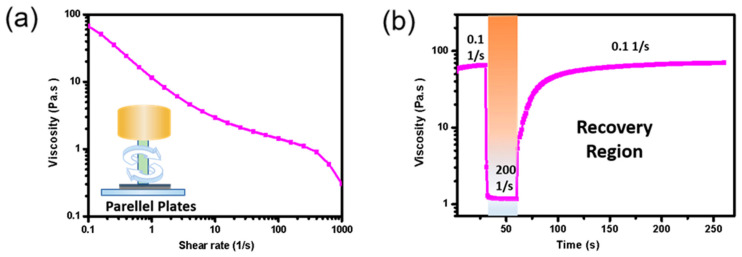
Rheological property of Ag/MWCNT composite: (**a**) shear thinning behavior with shear rate and (**b**) thixotropic behavior.

Figure 2a depicts the rheological behaviors of the Ag/MWCNT composite in terms of the printability of the ink in the screen printing process. The steady-state flow step measured by cone–plate rheometer reveals that the composites have shear thinning behaviors suitable for printing processes; shearing force induces disintegration of agglomerates or deforms the shapes of particles, resulting in reduced interactive forces between the particles [43]. We also measured the thixotropic behavior of the composite, whereby a reduction in structural strength with a shear loading and structure regeneration with time was set up to simulate the composite flow during the screen printing process. As shown in Figure 2b, the shear rate was kept at 0.1 s^−1^ for 30 s in the first interval and increased to 200 s^−1^ for 30 s in the second interval of the squeezing process. Then, the shear rate was brought back to 0.1 s^−1^ and held for 110 s in the third interval to observe leveling of the composite with time after finishing the printing. When the composites were under a considerable shear rate of 200 s^−1^ during the second interval, there was a decrease in viscosity, which then recovered with time (time dependency). Moreover, gradual recovery during the third interval was observed and the difference in recovery (%) between the initial point (60 s) to the end pint (110 s) was 80%. Since the recovery velocity determines the leveling time, which in turn determines the pattern quality, recovery times that are too short or too long should be avoided [44]. In this study, the Ag/MWCNT composite was suitable for high printability, since the composite recovery time was sufficient to allow a uniform pattern. In addition, the composite showed a high percentage of recovery of 80%, meaning the high elasticity allowed us to print 100 μm of a fine pattern.

**Figure 3:**
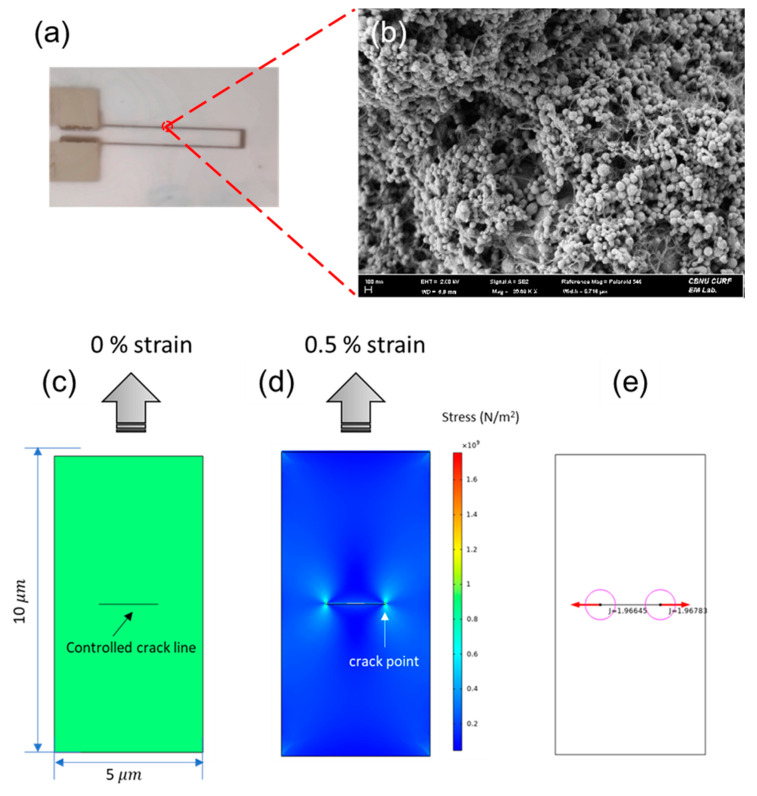
(**a**) Optical image of the printed wind sensor on PEN and its (**b**) SEM image. (**c**–**e**) Simulation of the crack with the J integral method obtained from COMSOL (ver.5.6).

Figure 3a shows optical images of Ag/MWCNT composite lines printed on PET with a line width of 100 μm. The composite lines exhibit sharp edges and display uniform and well-defined line widths. Moreover, no voids or disconnected line morphology were observed, which is important for highly stable and sensitive strain sensors. High sensing performance was also observed through the fully distributed MWCNTs with Ag nanoparticles, as shown in the SEM images. This structure was the basis of the piezoresistive sensing mechanism, which was investigated further using simulation software (COMSOL Multiphysics 5.6), as can be seen in Figure 3c.

This model shows the internal cracks in a rectangular solid subjected to the same strain (tension, in this case). In terms of the geometry, it was a rectangle measuring 10 × 5 µm with a crack measuring 50% of the width at the center. The crack was set perpendicular to the load direction, and the energy release rate at the crack tip was calculated using the J integral method [45]. When 0.5% strain was applied, a crack appeared in the perpendicular direction of the tension with two crack points, as can be seen in Figure 3d. The energy release rate of a crack extension along the current direction of the crack was calculated to be 1.96 (Figure 3e) and the value increased as the crack size increased. In the Figure 3e, J denotes the difference between the work applied by the external force and the energy used for the crack at the crack tip when the external force is applied to the crack body. Thus, the J value increases with increasing applied strain because some of the strain energy is consumed to create cracks. This simulation visually explains the occurrence of cracks that are difficult to identify in experiments in terms of the J integral energy.

**Figure 4:**
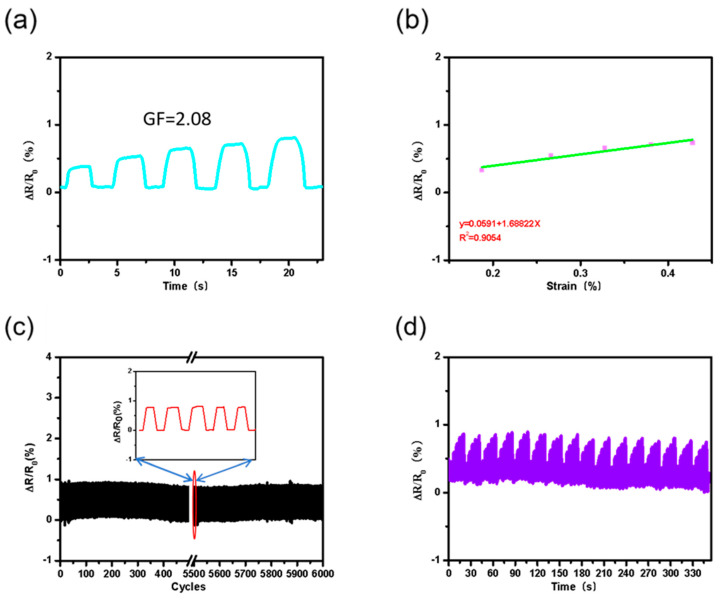
(**a**) A continuous variety of strains applied to the sensor. (**b**) continuous progressive input of different strain. (**c**) Durability of composites under 6000 cycles with low tensile deformation. Here, ∆R/R_0_ is a function of multiple bending and releasing cycles with 0.306% strain for Ag/MWCNT composites, where ∆R denotes the resistance differences under bending and release and R is the initial resistance in the relaxed state. (**d**) High stability of the sensor under continuous multiple strain cycles.

To investigate the sensing properties of the Ag/MWCNT composite printed on the PEN, we characterized the sensor with the applied strain under tension from 0.1% to 0.5% (Figure 4a). The gauge factor, defined as [G.F. = (ΔR/R_0_)/ε] [46,47], was calculated as 2.08 to determine the sensitivity of Ag/MWCNT composite strain sensors. Here, R_0_ denotes the resistance of the composite strain sensor without strain, ΔR denotes the resistance change under strain, and ε denotes the strain. This value is generally found in commercial strain sensors. In addition, we found the linearity of the sensor to be 90%, as shown in Figure 4b. To measure the response repeatability and durability according to line width, repeated stretching and release cycles were applied on the sensors. Figure 4c shows the resistance changes of the sensors, resulting in a fully recoverable electrical resistance without hysteresis at 0.306% of stretching for 6000 cycles. In addition to the cyclic stability test under a single strain, the high stability of the sensor was demonstrated under continuous and diverse strains (Figure 4d).

**Figure 5:**
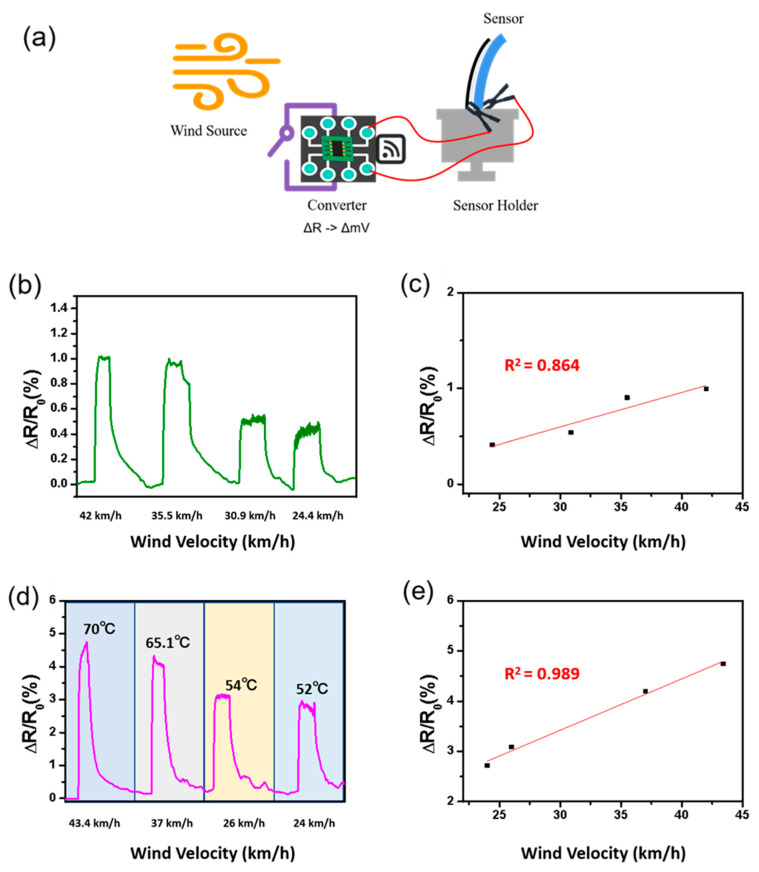
(**a**) The scheme of wind sensing system. (**b**) The effect of wind velocity on the resistance change. (**c**) The calculated linearity based on the result of the wind velocity change. (**d**) The effect of temperature on the resistance change. (**e**) The calculated linearity based on the results of the wind velocity changes under different temperatures.

To measure the wind sensing ability of the screen-printed sensor based on the wind velocity and temperature, the sensor was adapted to a home-made sensing system with a wind gauge, as can be seen in Figure 5a. The wind velocity range was from 24.4 km/h to 42 km/h, while the sensing sensitivity was expressed as an average value measured per minute for each level. Figure 5b reveals the effects of wind velocity on resistance changes, resulting in decreased resistance with wind velocity. Since lower wind velocity induces lower applied pressure to the surface of the flexible sensor, this leads to decreased bending pressure. Figure 5c shows the linearity calculation, with the result found to be 86%, based on the result of the wind velocity change. This is lower than the 90% obtained in the bending test, as shown in Figure 4a. Since the wind velocity causes sensor vibration during the measurement, stability issues may occur because of this side effect. We also investigated the sensing effect according to temperature change, using a range from 52 °C to 70 °C, at the same wind velocity shown in Figure 5d. As a result, the resistance change was increased to about 5 times higher than at room temperature due to the intrinsic properties of the metal hybrids. The linearity was 98%, as shown in Figure 5e, which was >10% higher than that of the wind velocity change at room temperature dur to the high thermal coefficient of Ag. These results prove that the sensor is also sensitive to the temperature effect with precise linear sensing features.

**Figure 6:**
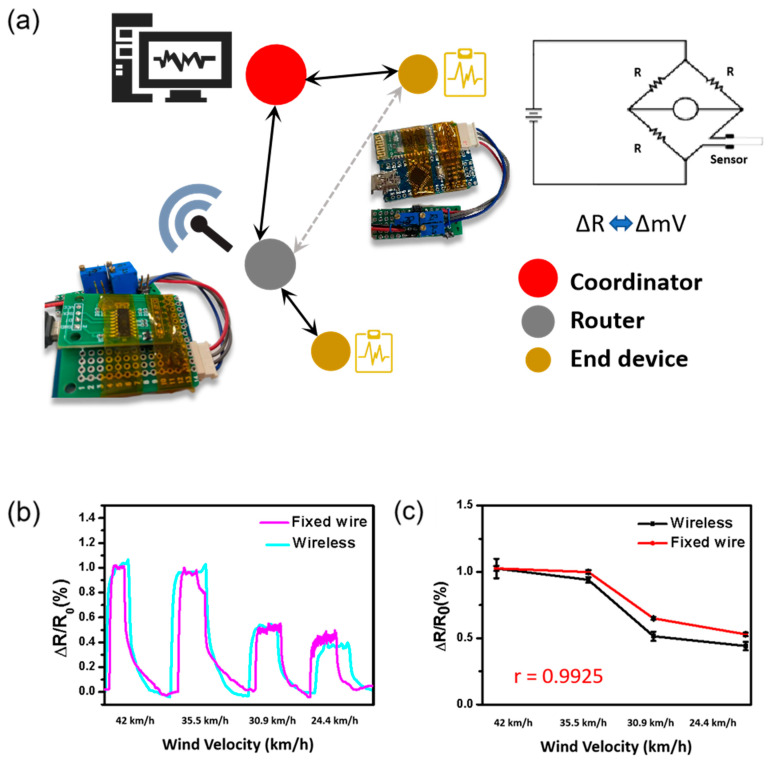
(**a**) The Wheatstone bridge design based a home-made IoT system, which is used to convert the real-time resistance change. (**b**,**c**) Synchronization of sensing responses between wireless and wired sensors for various wind velocities.

Figure 6 shows the wireless sensing responses based on the same experimental design as used in Figure 5. The Internet of Things (IoT) is flexibly applied in the conversion and transmission of electrical signals. The design of the Wheatstone bridge converts the real-time resistance change rate generated by the sensor under the influence of wind power into the voltage transformation rate and transmits data to the cloud through wireless devices so that coordinators can record and analyze data (Figure 6a). Different wind forces were applied to the sensor at room temperature to make the wind sensor produce the corresponding strain. Figure 6b shows a comparison analysis diagram obtained using the wireless sensor and fixed wire. Figure 6c shows 99% synchronization of the sensing response between a wireless and wired sensor under the same wind forces. The above demonstrate that the screen-printed sensor used in this study is of low cost and can be mass-produced through a simple printing process, as well as its high suitability for measuring the effects of wind velocity at various temperatures. In addition, this suggests that it is spatiality expanded because there is no restriction when the wind strength measurement requires the use of a wireless method.

## 4. Conclusions

In summary, we screen-printed a strain sensor based on Ag/MWCNT composite that can be used for measuring wind velocity at various temperatures. The rheological proper ties of the composites, assessed via flow curve test and thixotropic behavior, were investigated to confirm the high printability of the composite formula, resulting in shear thinning behavior and an 80% recovery ratio, which contributed to the high pattern fidelity during the printing process. After confirming the composite’s properties in the proper printing process, we investigated the sensing properties of the printed sensor using a bending test, resulting in a gauge factor (G.F.) of 2.08 with 90% linearity and high durability after 6000 bending cycles. In addition, the sensor was suitable for measuring wind velocity at temperatures ranging from room temperature to 70 °C, with 98% sensitivity, which was attributed to the intrinsic properties of the metal hybrids. Finally, we practically demonstrated that the performance of the IoT-based wireless sensor matched that of the wired sensor, making a case for the mass-production of low-cost screen-printed wind sensors. Compared to other recent works, the wireless screen-printable flexible strain sensor revealed higher performance in terms of stability and applicability with a simple production process. We believe its outstanding characteristics mean it will have wider application prospects.

## Figures and Tables

**Table 1:** Summary of the performance results for carbon-based flexible strain sensors.

Materials	Preparation Process	Stability	GF	Application	Ref.
Graphene/polyimide	Coating/Depositing	1500 cycles	0.96	Wind sensor	[38]
Carbon	Screen printing	No	3.27	health monitoring	[39]
CNT/polymer	Monte Carlo techniques	No	2	-	[40]
MWCNT/silicone rubber	Sub-surface printing of nanocomposite inside matrix	5 cycles	1.5	-	[41]
MWCNT/epoxy resins	Casting film	5 cycles	4.45	wearable electronics	[42]
Ag/CNT	Screen printing	6000 cycles	2.08	Wireless wind sensor	This work

## Data Availability

Not applicable.
